# A new species of *Tullbergia* (Collembola, Tullbergiidae) from Buenos Aires, Argentina

**DOI:** 10.3897/zookeys.416.6923

**Published:** 2014-06-16

**Authors:** José G. Palacios-Vargas, Ana E. Salazar Martínez

**Affiliations:** 1 Laboratorio de Ecología y Sistemática de Microartrópodos, Departamento de Ecología y Recursos Naturales, Facultad de Ciencias, Universidad Nacional Autónoma de México, 04510 México, D. F.; 2 División Entomología, Museo de La Plata, Paseo del Bosque s/n, 1900 La Plata, Argentina

**Keywords:** Taxonomy, Punta Lara, Buenos Aires

## Abstract

A new species of *Tullbergia* from Argentina is described and illustrated; it is differentiated from *Tullbergia paranensis* by the number of vesicles of postantennal organ, pseudocelli shape and its formulae and the number of dorsal sensilla on Ant. IV. In addition a key for the identification of the members of the family from Argentina is included.

## Introduction

Tullbergiidae is a small family with important diversity in the Southern Hemisphere, it has a total of 32 genera and 216 species in the world (Bellinger et al. 2013); but from Argentina only 15 species in 6 genera are known; six of them belong to *Tullbergia* (Bernava Laborde & Palacios-Vargas, 2008). These species have been found in Argentinian localities such as Buenos Aires, Entre Ríos, Neuquén and Córdoba, always associated to soils with high organic matter content. The lack of studies in other parts of the country could explain their absence in other Argentinian locations.

Tullbergiidae is an euedaphic group of small to very small size Collembola (0.4–1.5 mm except *Tullbergia antarctica* Lubbock, 1876 which is 3–4 mm) without pigmentation, eyes and furcula. They are very sensitive to ecological changes and so of importance for detecting the impact of different factors on soil ecology. Most important contributions to the taxonomy of the family in Argentina were done by Cassagnau and Rapoport (1962), Izarra (1965, 1969, 1972, 1975) and Rapoport (1962, 1963) while information about this and other families of Collembola from Argentina is summarized by Bernava Laborde and Palacios-Vargas (2008). In this paper we describe and illustrate a new species of Tullbergiidae which resembles *Tullbergia paranensis* Izarra, 1969.

## Materials and methods

Specimens were obtained from soils samples that were processed by Berlese-Tullgreen funnels and preserved in 75% alcohol. Later, slides were prepared in Hoyer’s solution. Specimens were studied and measured with a phase contrast microscope.

Abbreviations: Most of the abbreviations and terminology follows Dunger and Schlitt (2011). The provinces cited from Argentina are Bs As = Buenos Aires, Cba = Córdoba, E R = Entre Ríos, L P = La Pampa, Nq = Neuquén, Tuc = Tucumán.

## Taxonomy

### Tullbergia

Taxon classificationAnimaliaPoduromorphaTullbergiidae

Lubbock, 1876

#### Diagnosis.

Body white (without pigment); Ant. IV without an extremely large apical papilla. Tibiotarsi without clavated setae (seldom few weakly developed ones); Ant. III organ with a maximum of 5 elements including 2-3 large sensory clubs, two of them bent towards one another; Abd. VI without crescentic ridges and additional dorso-lateral spines. Th. I-III with 1, 1-2, 1-2 pseudocelli of type I (after Weiner and Najt 1991). PAO with 40-50 (seldom more) rod-like vesicles in two rows; Ant. III organ with a well visible protecting fold.

#### Type species.

*Tullbergia antarctica* Lubbock, 1876

### Tullbergia alcirae sp. n.

Taxon classificationAnimaliaPoduromorphaTullbergiidae

http://zoobank.org/9D213EA9-95C4-4C94-9B5A-5E0CDB0E1357

Figs 1 –8

#### Material examined.

Type locality: Argentina: Bs As: Punta Lara, 34°48'S; 58°00'0"W, ex soil, grassland dominated by grasses (Poaceae) and sedge (Cyperaceae), May 2010. The specimens were extracted from soil samples in Berlese’s funnels. A. Salazar Martínez collector.

Holotype. Female mounted on slide. Original label: 5404/1 deposited at Colección División Entomología, Museo de la Plata. Paratypes: 2 females paratypes, 2 males paratypes, all of them mounted on slides. Original label: 5404/2-4 deposited at Colección División Entomología, Museo de la Plata. 6 females, 4 males and 11 juvenil paratypes with the same collecting data are kept at senior’s author institution with catalog number 2436.

#### Diagnosis.

Ant. III organ with 3 thick curved dorsal sensilla, one ventral sensillum; 6 dorsal sensilla on Ant. IV; about 72 vesicles on postantennal organ; pseudocellar formulae 11/122/22221: one minute empodial appendix, two slightly clavate tenent hairs ventrally on each tibiotarsus.

#### Description.

Body length (n=12) 1.2 mm (range 0.83–1.4 mm), with moderately long macrosetae 61 µm (range 56–66), and short microsetae 18 µm (range 15–23), all of them smooth and thin. Relatively uniformly distributed fine intergumentary granules, interspersed with somewhat coarser granules on Abd. VI. Antennal bases well delimited.

**Figures 1–4.**
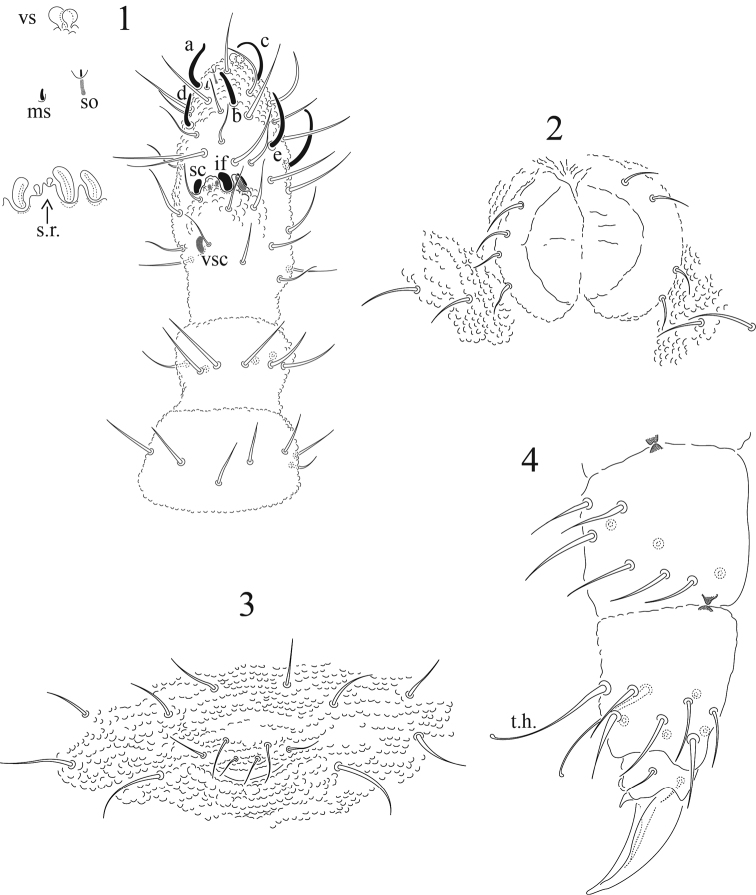
*Tullbergia alcirae* sp. n. **1** antennal segments I to IV with details of sensorial structures **2** ventral tube **3** female genital plate **4** femur and tibiotarsus III.

**Figures 5–8.**
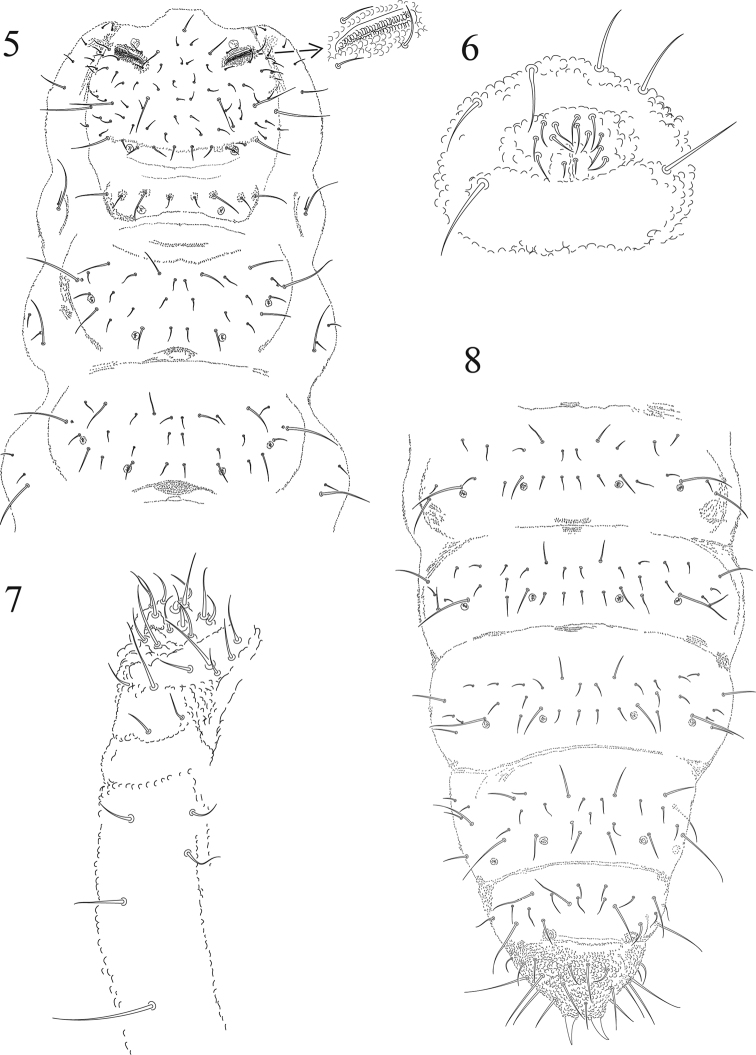
*Tullbergia alcirae* sp. n. **5** dorsal head and thoracic chaetotaxy **6** male genital plate **7** right half of labial and postlabial quetotaxy **8** dorsal abdominal cheatotaxy.

Ratio head: antenna = 1: 0.8. The relative lengths of Ant. I: II; III; IV are as 1: 1.6; 1.9; 1.8. Ant. III and IV fused dorsally (80 µm). Sense organ of Ant. III with two small sensory rods concealed behind one of the two integumentary folds; in addition there are three thick sensilla clubs, which are rounded at tip; two strongly bend towards each other and not concealed by cuticule. At bases of cuticle, the third sensillum concealed by one tegumentary fold. There is a ventral straight sensillum protected by three setae longer than other ventral setae. Ant. IV furnished with 6 sensilla, 3 of them very thin and long, three thick and short, one distinct subapical pit, one microsensillum and one slightly trilobed apical bulb (Fig. 1). Postantennal organ elliptical 35 µm (range 28–51), as long as width of the Ant. I, consisting of 72 (65–86) simple vesicles lying in two regular rows (Fig. 5).

Legs chaetotaxy from I to III, coxae, 2,7,7; trochanters 5,5,5; femora, 9,9,9 (each with one ventral acuminate tenent hair); tibiotarsi (13,13,12), each with 2 ventral slightly clavate tenent hairs). Tibiotarsi short, 28 µm (range 22–31). Pretarsus with two setae each. Ungues untoothed. Empodial appendage rudimentary, in shape of a minute claw-like process (Fig. 4). Dorsal pseudocelli on the body arranged as follows: 11/122/22221. The pseudocelli are crescentic with 4–5 rows of granulations (type III of Weiner and Najt 1991).

Dorsal chaetotaxy of thorax and abdomen in Figs 5 and 8 and Table 1. Th. II and III with one lateral microsensillum on each side. Dorsal chaetotaxy of abdomen on Fig. 8. Head ventrally mainly with microsetae, three pairs of postlabial setae, posterior one is macroseta (Fig. 7). Th. II and III ventrally with one pair of setae each and four setae of different sizes on each pleural side.

Ventral tube with 6 + 6 setae (4 + 4 distal and 2 + 2 basal setae, Fig. 2). Pseudocelli of Abd. V are guarded by sensory setae similar to normal setae. Abd. IV tergite without a surrounding semicircular narrow ridge. Two anal spines relatively short, 37 µm (range 34–42) and weak, but usually slightly curved and placed on high papillae which touch at their bases (Fig. 8). Ratio anal spine: unguis III: 1: 1.5. Genital female plate with 3 pairs of pregenital setae, 2 pairs of circumgenital and one pair of eugenital setae. Males with 3 pairs of pregenital setae, 6 pairs circumgenital setae and 1 + 1 eugenital setae (Fig. 6).

**Table 1.** Dorsal chaetotaxy of *Tullbergia alcirae* sp. n.

	Thorax				Abdomen			
row	I	II	III	I	II	III	IV	V
a	-	8	10	10	12	12	10	8
m	-	8*	8*	2**	2**	2**	2***	8
p	8	10	10	10	12	12	10	4
pl	2	3	3	2	3	3	4	2

* Missing m2,

** m4 present,

*** one medial setae on row m.

#### Etymology.

The new species is named in memory of Dr. Alcira Bischoff, soil fauna researcher from Facultad de Ciencias Naturales y Museo of Buenos Aires, Argentina.

**Distribution.** Known only from Punta Lara, the type locality in Buenos Aires Province, Argentina.

#### Ecology.

*Tullbergia alcirae* sp. n. was found in soil samples. The specimens were taken from the first 10 cm in horizon A, with a density of 624 ± 30 individuals per m^2^. Fredes et al. (2009) have pointed *Tullbergia* sp. as a good trampling indicator in a recreation area from Miramar, Buenos Aires, Argentina. *Tullbergia* species in Argentina come from an extensive area with different environmental conditions but the low sampling intensity doesn`t allow to develop any biogeographic hypothesis about its distribution.

#### Discussion.

*Tullbergia alcirae* sp. n. is similar to *Tullbergia paranensis* because they share the presence of 3 thick curved sensilla on Ant. III, isolated by digitations of the tegumentary fold, and also by the presence of one ventral sensillum and the same shape of pseudocelli. *Tullbergia alcirae* sp. n. differs from *Tullbergia paranensis* in having more vesicles in the postantennal organ (72 *vs.* 30), the presence of a minute empodial appendix (versus none). It also differs in the pseudocellar formulae (11/122/22221 vs /111/11111). The number of dorsal sensilla on Ant. IV is also different (6 vs. 5). *Tullbergia* is supposed to have pseudocelli of type I (Weiner and Najt 1991), but it is of type III in these two species, pointing to the need of a revision of the Tullbergiidae from Argentina.

#### Variation.

Tenent hairs are very slightly capitated and often leg one has one capitated and one acuminate tenent hairs.

### Key to species of Tullbergiidae from Argentina (with provinces where they are distributed)

**Table:** 

1	No pseudocelli, pigment, eyes, unguiculus or furcula present	*Pachytullbergia scabra* Bonet, 1947 [Nq]
1’	With pseudocelli on head and body	2
2	Sensilla of Ant. III are completely free and not protected by one cuticular fold	3
2’	Sensilla of Ant. III protected by one cuticular fold	4
3	Postantennal organ is round and with about 60 vesicles	*Tullbergiella*. *..* 6
3’	Postantennal organ elliptical, with more than 60 vesicles	*Tullbergia*...10
4	Abd. VI dorsally with 6 or 8 strong spines, or spine-like processes	*Dinaphorura*...9
4’	Abd-VI dorsally with only 2 or 4 spines	5
5	True pseudocelli present, there are 4 spines dorsally on Abd. VI	15
5’	No real pseudocelli present, only 2 spines on Abd. VI	*Mesaphorura*...7
6	Ant. IV with four sensilla, pseudocelli on all abdominal segments	*Tullbergiella allendei* Izarra, 1975 [Bs As, LP]
6’	Ant. IV with three sensilla, pseudocelli absent on Abb. II and III	*Tullbergiella humilis* Izarra, 1965 [Bs As]
7	Abd. IV seta P1 is a microseta	*Mesaphorura iowensis* (Mills, 1932) [Bs As]
7’	Abd. IV seta P1 is a macroseta	8
8	Abd. IV seta m5 present, Abd.VI seta l’2 present in the anal lobe	*Mesaphorura macrochaeta* Rusek, 1976 [Bs As]
8’	Abd. IV seta m5 absent, Abd.VI seta l’2 absent	*Mesaphorura krausbaueri* Börner, 1901 [Bs As, Cba, E R, Tuc, L P, Nq]
9	PAO elongated with 22–24 vesicles, apical bulb of Ant. IV with 2 or 3 lobes, maximum length 1.8 mm	*Dinaphorura spinosissima* (Wahlgren, 1906) [Nq]
9‘	PAO triangular with 12–15 vesicles, apical bulb of Ant. IV mushroom-shaped, maximum length 1.1 mm	*Dinaphorura americana* Rapoport, 1962 [Bs As, Nq]
10	Abd. I-IV, at most with one pair of pseudocelli on each side, Ant. IV with 3-5 sensilla	11
10’	Abd. I-IV, with two pairs of pseudocelli on each side	12
11	Th. I without pseudocelli, OPA with 40–50 vesicles in three or four rows	*Tullbergia inconspicua* Izarra, 1965 [Bs As]
11’	Th. I with one pair of pseudocelli, OPA with 30 vesicles	*Tullbergia paranensis* Izarra, 1969 [E R]
12	Ant. IV with six sensilla	*Tullbergia alcirae* sp. n. [Bs As]
12’	Ant. IV with less than six sensilla	13
13	Ant. IV with 4 sensilla, OPA with more than 100 vesicles	*Tullbergia trisetosa* (Schäffer, 1897) [Nq]
13’	Ant. IV with 5 sensilla, OPA with less than 70 vesicles	14
14	Abd. IV with 4 setae between anterior pseudocelli. OPA with 30-50 vesicles	*Tullbergia meridionalis* Cassagnau & Rapoport, 1962 [Bs As, Cba, E R, Nq]
14’	Abd. IV with 2 short setae between anterior pseudocelli. OPA with 34-63 vesicles	*Tullbergia ventanensis* Rapoport, 1963 [Bs As]
15	Abd. VI with four real posterior anal spines of the same size, PAO with 60 vesicles in two rows	*Anaphorura lavadoi* Izarra, 1972 [R N]
15’	Abd. VI with 1 + 1 dorsolateral small spines in front of the posterior pair of anal spines, PAO with (38) 40–(45)50 vesicles in 2–3 rows	*Stenaphorura quadrispina* (Börner, 1901) [Bs As]

## Supplementary Material

XML Treatment for Tullbergia

XML Treatment for Tullbergia alcirae
